# Progress in Medicine

**Published:** 1897-09-18

**Authors:** 


### Progress in Medicine.

### DIABETES.

**Tests for Sugar.**—R. T. Williamson[1] has devised a simple method of diagnosis where urine cannot be obtained. It is based on the power diabetic blood has of decolourising solutions of methyl blue. A few drops of blood are obtained from the finger, and 20 cub. millimetres of it are added to 40 of water, and 1 cub. centimetre of a 1 in 6,000 solution of methyl blue in water. Finally 40 cub. millimetres of liq. potassæ are added, and the tube containing the mixture is placed in a vessel of boiling water. In four minutes the blue changes to a yellow colour if diabetes is present. This test may also be applied to urine. A rapid quantitative method of discovering the sugar in the urine is given by Duhomme[2]. He puts 20 drops of Fehling's solution into a test tube, boils and adds the urine drop by drop until complete decolourisation has taken place. Let 9 be the number of drops required, then 100 divided by 9 expresses the amount of sugar in grains per litre. If we multiply this by $\frac{3}{7}$ we have the grains per ounce ($\frac{100}{9} \times \frac{3}{7} = 4{\cdot}7$ grains). To save time still further he uses six tubes, adding one drop to the first, two to the second, three to the third, and so on, boiling each in succession, and if there is no result then six more drops to each tube, which gives seven, eight, nine, &c., drops in each. Schreiber's sugar reagent is formed by dissolving in 100 c.c. of distilled water, two grams each of cupric sulphate and sodic salicylate with eight grams of crystallised sodic carbonate, and filtering. If five cubic centimetres are boiled with diabetic urine a yellow precipitate and a glaze on the tube are produced. The quantity present can be estimated by noting that, if 2 c.c. of urine are required to produce the yellow glaze, there is 10 per cent. present; if 2·5, there is 1 per cent.; and if 3 must be added, there is one-tenth per cent. only.

In cases where there is any doubt as to the results of Fehling's test, R. T. Williamson[3] recommends the following: Sodium acetate is put into a test tube till half an inch deep, then an equal quantity of phenyl hydrazin also in powder, and the tube is half-filled with urine. This is boiled for two minutes. If there is no precipitate the substance which reduced the Fehling was not sugar. Sugar gives a yellow deposit which, seen under the microscope, is made up of sheaves of acicular crystals. Lactose yields small yellow globules with very short spines, and glycurosic acid a very scanty deposit like sugar.

Another method of diagnosis from the action of stains on the blood is recommended by L. Bremer.[4] He spreads a drop of blood over a slide and heats it to a temperature of 135 deg. C. for six or ten minutes. The slide is then placed in a bath of Congo red, methylene blue, or the Ehrlich-Biondi stain for one and a half minutes. In the two first, diabetic blood is colourless and normal blood red, while the other gives orange as opposed to violet. Similarly with diabetic urine. If a mixture of powdered eosin and gentian violet be added to a test tube of diabetic urine and heated gently, a purple or blue cloud will form near the surface of the urine.[5] There are certain fallacies in the use of this test, such as the reaction presented also by diabetes insipidus.

**Pathology and Complications.**—Pavy[6] gave a statement of his present views before the Glasgow Med.-Chirurgical Society, in which he considered the difference between the healthy man and the diabetic. The cause of this is the presence of grape-sugar in the blood of the latter. This is normally prevented by the change of the carbohydrates into fat and a proteid substance in the intestinal villi and in the liver. In the glycosuria of old men the capacity for conversion has declined, while in true diabetes there is a graver defect in the mechanism. The treatment so far can only be abstinence from carbohydrate diet until the converting power is regained. This recovery is aided by codeia, and he warns us that many diabetic breads are unreliable, and that toast is actually worse than bread, because much of the starch is changed to dextrine, which facilitates the absorption of carbohydrates from the intestine. When a patient is passing no sugar under a rigid diet, and, having gained weight, begins to lose it again, it is time to try carefully a return to carbohydrate food. The diabetes of young adults is probably due to a ferment which breaks up the tissue proteids themselves into glucose and other substances. It is thus a distinct malady.

In a previous paper he showed that in experimental phloridzin diabetes the ingestion of this substance, derived from cherry bark, or even of phloretin, which is the sugar free part of it, produces a great temporary glycosuria, and, moreover, sugar is for a time present in the blood to an abnormal amount, which had been denied by Von Mering and others. His views are strongly opposed by Klemperer, who regards the glycosuria as dependent on a condition of the renal epithelium.

Loeb[7] believes that many persons pass small quantities of sugar for a long period without any of the other symptoms of diabetes. Some of these cases he has found in later years with the fully-developed disease, though there are others who recover entirely. Among the early symptoms are neuralgia, anosmia, and cramps in the legs. Brissac[8] records six cases of recurrent transitory diabetes. The amount varied much, diminished rapidly under dietary, and was less in later attacks than in the first one, though the attacks lasted longer with intervening months or years of perfect health. There was generally a temporary albuminuria and a large amount of uric acid. Some of these cases are gouty, and some neurotic. They may go on to chronic diabetes or be forerunners of different diseases.

Alimentary glycosuria generally occurs in healthy persons after eating 150 grams of glucose at once. Strümpell[9] found no excretion took place in marasmic individuals suffering from organic disease or from chlorosis, gout, cirrhosis of the liver, and certain diseases of the nervous system. In corpulent beer drinkers it was easily produced. Strauss[10] examined the production of this glycosuria in 250 persons suffering from nerve disease, and found it appeared readily in 36 per cent. of traumatic neuroses, in 70 per cent. of cases of delirium tremens, 55 per cent. of lead poisoning, and 9 per cent. of organic brain disease, but the thyroid gland appears to have little influence on the production of this alimentary glycosuria. Albu, indeed, lays down that it never occurs where renal sclerosis exists, and Strauss failed to produce it in any cases of cancerous cachexia, though their blood contains more than the normal amount of sugar. In his experiments he used grape sugar only, as cane sugar is more easily broken up in the body. It does not appear, however, from these results that easily produced glycosuria can be taken as a test for the existence of certain nerve diseases, as has been suggested by previous writers. Bettman[11] believes that some relation does exist between Grave's disease and diabetes, though the number of the cases where they have occurred together is very small, and the bulbar theory put forward to explain it is not proved. He would look on the diabetes as a secondary effect of the poison of the other disease on the body metabolism. He refers to seventeen such cases, and believes that a true diabetes of the severe type may thus arise.

The disappearance of the knee jerks in diabetes is only to be found in some 7 per cent., according to Grübe, but R. T. Williamson and others gave a much larger percentage. Williamson[12] has now examined another series of 50 cases, and finds that, as before, the reaction is absent in half the number. He accounts for the difference as due to the number of severe diabetic cases in young people among his patients. In early stages, indeed, the jerks may be obtained, though they vanish as the disease becomes graver in an increasing number of cases. Changes in the cord and peripheral neuritis do exist in a few of them, but in many others there are no histological changes to be found. In the last days of life and in coma the great majority of patients lose the jerks, while in the mild diabetes of aged well-to-do persons the loss is rarely noticed. It is well known that transient diabetes does sometimes appear in the course of general paralysis, and may even be an early symptom of it, but De Holstein[13] shows that long-continued diabetes may occasionally produce all the symptoms of general paralysis, which, however, may disappear on a strict diet. Thus, a man without any alcoholic or syphilitic history, who had suffered for twenty years from diabetes, developed all the symptoms of general paralysis, lost all mental control, threw away his fortune, and passed into an apathetic state; but finally recovered his former health, vigour, and mental habitudes when the sugar was got rid of, so much so that he again earned his living in a commercial business.

**Treatment.**—Munson[14] has, in perhaps an exaggerated manner, endeavoured to show the danger of eliminating carbohydrates from diabetic diet, especially in the causation of cerebral symptoms. In severe cases, on a pure flesh diet, sugar is still formed from the breaking up of proteids. Now, such a diet means increased metabolism, which is the defect of diabetes; and to remove carbohydrates, which retard metabolism, increases the rapidity of the downward course towards asthenia, and also multiplies the production of toxins. Hogarth[15] notices a case of violent stabbing pains in the epigastrium, generally occurring in the early morning, with some tenderness on deep pressure in the right hypochondriac region. The patient had been taking saccharin to the amount of three grains daily for some years, which is said to cause, at times, neuralgia of the solar plexus. At any rate, a complete cure followed his giving up the saccharin. A milk diet has been of late considered harmful, though Saundby and others allow twenty or thirty ounces daily. Bouchardet only gives it in albuminuria, impending coma, or other complication, on account of the change of lactose into glucose in many diabetics. Œttinger[16] believes that many of them do not change it, just as many of them do well on inulose or mannite, and shows that milk is invaluable for some of these patients, and its administration must depend on the idiosyncracy of each person. Gilbert and Carnot[17] give as a remedy an extract of fresh pigs' liver, and report considerable success. Of 13 cases, three showed temporary diminution of the sugar, five improved permanently, and in four the glycosuria completely ceased. The urea and uric acid are increased while the liver extract is being taken, but the effect on the polyuria is more doubtful. The results are most marked in cases of hepatic origin where enlargement or cirrhosis of the liver is present, unless that organ is too far deteriorated. Thus obese diabetics passing over 17 oz. of sugar daily only improve temporarily. The extract is made by mincing 3 oz. to 5½ oz. of fresh liver, macerating two hours in half a pint of water at 95 deg. to 100 deg. Fah., then filtering and expressing the juice. The liquid can be given as an enema, or by mouth in divided doses, but rectal administration is most successful.

Saundby[18] first places his patients on a rigid diet, and when the amount of sugar is reduced as low as possible and the general condition improved after, say, a week's trial, he adds a baked potato to the daily diet for another week, and if there is then no relapse two are permitted. Gluten breads, he finds, are most unsatisfactory, and may contain as much starch as ordinary ones. Almond and cocoanut flour should alone be used for bread, cakes, and other purposes; and neither these, gluten or soy flours should ever be given unless previously analysed, as numerous adulterated samples are in the market. The iodine and Fehling's tests should be employed by every practitioner to safeguard his patients. When digestive disorders and acetonuria show the likelihood of coma, Teschemacher[19] gives purgative enemata of 10 to 40 grammes of glycerine in half a litre of water retained for half an hour two or three times a week with excellent results. Rectal feeding for a time is also desirable, and aids the success of the treatment. Lepine[20] advises in coma the intra-venous injection of large quantities, even two or more litres of alkaline solution (10 grammes of sodium bicarbonate and seven of chloride to the litre), as early as possible. Large doses given by the mouth are not always absorbed, and indeed the prognosis still remains most unsatisfactory.

[1] Med. Rec., Feb. 6. [2] Med. Rec., March 27. [3] B. Med. Jour., March 13. [4] B. Med. Jour., Aug. 28. [5] Med. Rec., March 13. [6] Glasgow Med. Jour., Feb. [7] Am. Jour. Med Sc., March; and B. M. Jour., March 20. [8] B. M. Jour., March 13. [9] Birmingham Med. Rev., July. [10] Med. Week, April 9. [11] B. Med. Jour., March 6. [12] Lancet, July 17. [13] Med. Week, June 25. [14] Med. Rec., June 12. [15] B. Med. Jour., March 20. [16] B. Med. Jour., May 22. [17] Med. Week, May 10. [18] Allbutt's System, vol. iii. [19] Med. Week, April 9. [20] Med. Week, April 2.

